# Regionalization of the axial skeleton in the ‘ambush predator’ guild – are there developmental rules underlying body shape evolution in ray-finned fishes?

**DOI:** 10.1186/1471-2148-13-265

**Published:** 2013-12-05

**Authors:** Erin E Maxwell, Laura AB Wilson

**Affiliations:** 1 Paläontologisches Institut und Museum, Universität Zürich, Zürich, Switzerland; 2 School of Biological, Earth, and Environmental Sciences, University of New South Wales, Kensington, NSW 2052, Australia

**Keywords:** Actinopterygii, Axial skeleton, Modularity, Axial elongation, Saurichthyidae, Aulostomidae, Lepisosteidae, Esocidae, Beloniformes, Sphyraenidae

## Abstract

**Background:**

A long, slender body plan characterized by an elongate antorbital region and posterior displacement of the unpaired fins has evolved multiple times within ray-finned fishes, and is associated with ambush predation. The axial skeleton of ray-finned fishes is divided into abdominal and caudal regions, considered to be evolutionary modules. In this study, we test whether the convergent evolution of the ambush predator body plan is associated with predictable, regional changes in the axial skeleton, specifically whether the abdominal region is preferentially lengthened relative to the caudal region through the addition of vertebrae. We test this hypothesis in seven clades showing convergent evolution of this body plan, examining abdominal and caudal vertebral counts in over 300 living and fossil species. In four of these clades, we also examined the relationship between the fineness ratio and vertebral regionalization using phylogenetic independent contrasts.

**Results:**

We report that in five of the clades surveyed, Lepisosteidae, Esocidae, Belonidae, Sphyraenidae and Fistulariidae, vertebrae are added preferentially to the abdominal region. In Lepisosteidae, Esocidae, and Belonidae, increasing abdominal vertebral count was also significantly related to increasing fineness ratio, a measure of elongation. Two clades did not preferentially add abdominal vertebrae: Saurichthyidae and Aulostomidae. Both of these groups show the development of a novel caudal region anterior to the insertion of the anal fin, morphologically differentiated from more posterior caudal vertebrae.

**Conclusions:**

The preferential addition of abdominal vertebrae in fishes with an elongate body shape is consistent with the existence of a conservative positioning module formed by the boundary between the abdominal and caudal vertebral regions and the anterior insertion of the anal fin. Dissociation of this module is possible, although less probable than changes in the independently evolving abdominal region. Dissociation of the axial skeleton-median fin module leads to increased regionalization within the caudal vertebral column, something that has evolved several times in bony fishes, and may be homologous with the sacral region of tetrapods. These results suggest that modularity of the axial skeleton may result in somewhat predictable evolutionary outcomes in bony fishes.

## Background

With over 30 000 extant species, actinopterygian (ray-finned) fishes comprise almost half of all species of living vertebrates [1], and are also well represented in the fossil record. Actinopterygians show an enormous disparity in body shape associated with feeding and locomotor adaptations, which is correlated with the evolutionary and ecological success of the group [2]. Understanding how these shape changes arose provides insights into aspects of function and development underlying diversity patterns. Axial elongation is one aspect of the vertebrate body plan that has received a great deal of research attention, and has evolved convergently numerous times in both ray-finned fishes and tetrapods (e.g., [3-6]).

Two classical fish body plans [sensu 2], the anguilliform shape and a more rigid shape associated with ambush predation, are considered to be elongate [7]. Body elongation in fishes has been attributed to several factors, including increase in the number of vertebrae, increase in the length of vertebrae, increase in the length of the skull, and decrease in the depth of the body (reviewed by [4]). An additional mechanism, duplication of elements within a somite, has been proposed for some non-teleost actinopterygians [8]. A detailed investigation into axial elongation in actinopterygians found that the addition of vertebrae occurred in either the abdominal region, the caudal region, or both, and suggested these body regions were capable of independent evolution, being organised into separate developmental modules [3]. Modules are defined as subsets of traits that are tightly integrated due to shared developmental history or function [9]. The presence of weak interconnection between traits parcelled into different modules has been hypothesized to facilitate morphological evolution because it mitigates the widespread effect of constraints in a system and allows modules to vary independently (e.g., [10-12]). The evolutionary significance of modularity is twofold, reflecting within- and across-module connections. First, the high levels of connectedness within modules (integration) may constrain trait variation in a single module, essentially reducing potential for evolutionary change in some directions of phenotypic space [13]. Second, because there are weak connections across modules there is little interference between the adaptation of different functions, thus allowing modules to evolve toward their selected optima, favouring evolvability. Thus, changes between the abdominal and caudal modules of the fish axial skeleton should be more probable than disintegration of either of these modules.

**Figure 1:**
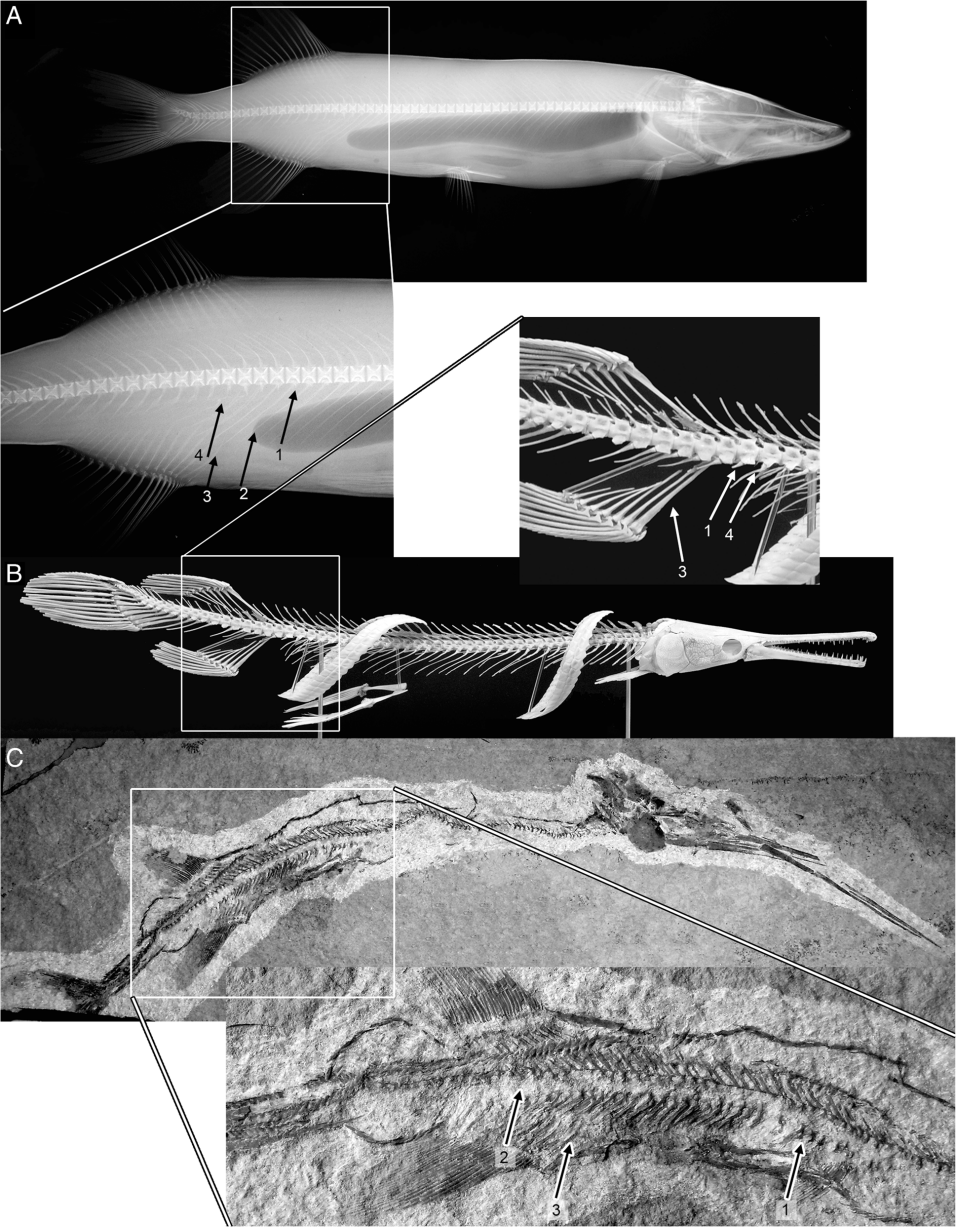
**The structure of the posterior abdominal and anterior caudal region in three elongate fishes showing the ‘ambush predator’ body shape. A**, *Esox lucius* (Esocidae); **B**, *Lepisosteus osseus*; **C**, *Saurichthys* cf. *paucitrichus* (PIMUZ T 534). The region around the abdominal-caudal transition is indicated in the insets, and the following morphological landmarks are indicated: 1, first vertebra bearing a haemal arch (i.e., first caudal vertebra); 2, end of the body cavity, 3, first pterygiophore of the anal fin; 4, last rib-bearing vertebra; 5, morphological transition between the anterior and posterior caudal regions as indicated by the reduced haemal arches and transition to T-shaped neural arches. Photo in panel B copyright PIMUZ, courtesy of T. Scheyer.

In their landmark study, Ward and Brainerd [3] pooled both elongate fish ecomorphotypes into a single category. However, subsequent studies focusing on elongation of the body in eels (Anguilliformes) have revealed that even at the ordinal level, changes in vertebral number may occur in any region [4], and may not be closely linked to the formation of an elongate body plan [14]. In this contribution, we ask whether distantly related groups of fishes showing morphological convergence in body shape are characterized by predictable changes in vertebral counts in the abdominal and caudal modules. In order to investigate this question, we selected the ‘ambush predator’ shape class (Figure 1). This body plan first became widespread among the saurichthyid fishes (Figure 1c) in the Lower Triassic (around 250 MYA), and the ecomorphotype has been occupied almost continuously since this time (e.g., [15,16]). In the modern ichthyofauna, this body plan is found in such distantly related fishes as Lepisosteidae (gars; Figure 1b), Belonidae (needlefish), Esocidae (pikes; Figure 1a), Sphyraenidae (barracudas), Fistulariidae (cornetfishes), and Aulostomidae (trumpetfishes).

Characteristics of the ‘ambush predator’ body plan include antorbital elongation of the jaws, and distinct, posteriorly displaced dorsal and anal fins [2]. It has been suggested that this body shape is less relevant to locomotor parameters than to a piscivorous diet [17,18]. The posteriorly displaced dorsal fin is suboptimal for acceleration performance [19], but is thought to delay the escape response of prey by giving the illusion that the predator is further away, thus decreasing striking distance [20] (looming effect: [21]). Thus, the posterior displacement of the median fins is a key feature of the body plan.

Changes in the relative position of morphological landmarks along the anterior-posterior axis of the vertebrate body are determined by patterning genes acting early in embryogenesis. The location of the abdominal–caudal transition is correlated with the expression of Hox genes in the paraxial mesoderm, with the anterior expression limit of Hox11-12 marking the positional onset of haemal arch-bearing vertebrae of both chondrichthyan [22] and teleost fishes [23], as well as the sacral–caudal transition in tetrapods [24]. The position of the posterior dorsal and anal fins also appears to be specified through the anterior expression of Hoxd12 in the developing dorsal and ventral finfolds [25]. The position of the posterior dorsal fin and the anal fin are dissociated in many fishes, but have been hypothesized to form a developmental module together with the anus [26]. The anus is generally, but not exclusively [27], located at the end of the body cavity at the boundary between the abdominal and caudal vertebral types, and constrains the anteriormost point of insertion of the anal fin pterygiophores. Hence, shared underlying patterning mechanisms and pre-existing hypotheses of modularity allow us to predict that the posterior displacement of the dorsal and anal fins in the elongate ‘ambush predator’ body type will be accomplished by the preferential addition of abdominal vertebrae.

Here, we examine changes in the number of abdominal and caudal vertebrae in seven actinopterygian clades, two non-teleost and five teleost groups. Unlike previous analyses on regionalized vertebral counts in fishes [3,4,14], we include fossil species in the analysis. This approach is particularly critical to examine elongation of the axial skeleton in gars (Lepisosteidae) which have few extant representatives but a rich fossil record, as well as the completely extinct Saurichthyidae, the first group of fishes to successfully exploit this ecomorphotype. We report that abdominal vertebrae are added preferentially in the majority (5/7) of the clades examined, and the number of abdominal vertebrae was significantly associated with increasing axial elongation in 3/4 of the clades in which this was tested.

## Results

### Chondrosteans

**Figure 2:**
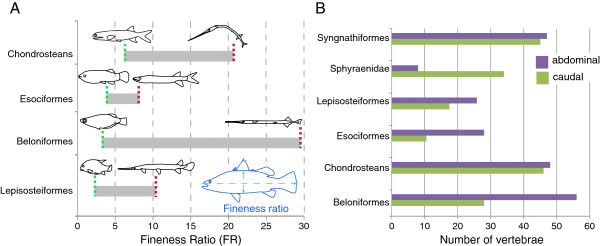
**Overview of variability in Fineness Ratio and regional vertebral count data within the analyzed data sets. A**, Range of Fineness Ratio (FR) values calculated for species sampled within the chondrostean, esociform, beloniform, and lepisosteiform data sets, including outgroup taxa. Species with the smallest and largest values for FR are illustrated for each clade: *Birgeria groenlandica* and *Saurichthys striolatus* (chondostreans); *Callipurbeckia minor* and *Lepisosteus osseus* (Lepisosteiformes); *Palaeoesox weileri* and *Esox tiemani* (Esociformes); *Oryzias profundicola* and *Pseudotylosurus* (Beloniformes). FR measures the relationship between body length and depth (right, inset). **B**, Bar graph illustrating the range in abdominal and caudal vertebral counts for species sampled within each dataset, including outgroup taxa. Note the greater range of abdominal counts than caudal counts in the lepisosteiform, beloniform and esociform data sets. The large range of caudal counts seen in the ‘Sphyraenidae’ data set stems from the inclusion of Pleuronectiformes as an outgroup.

**Figure 3:**
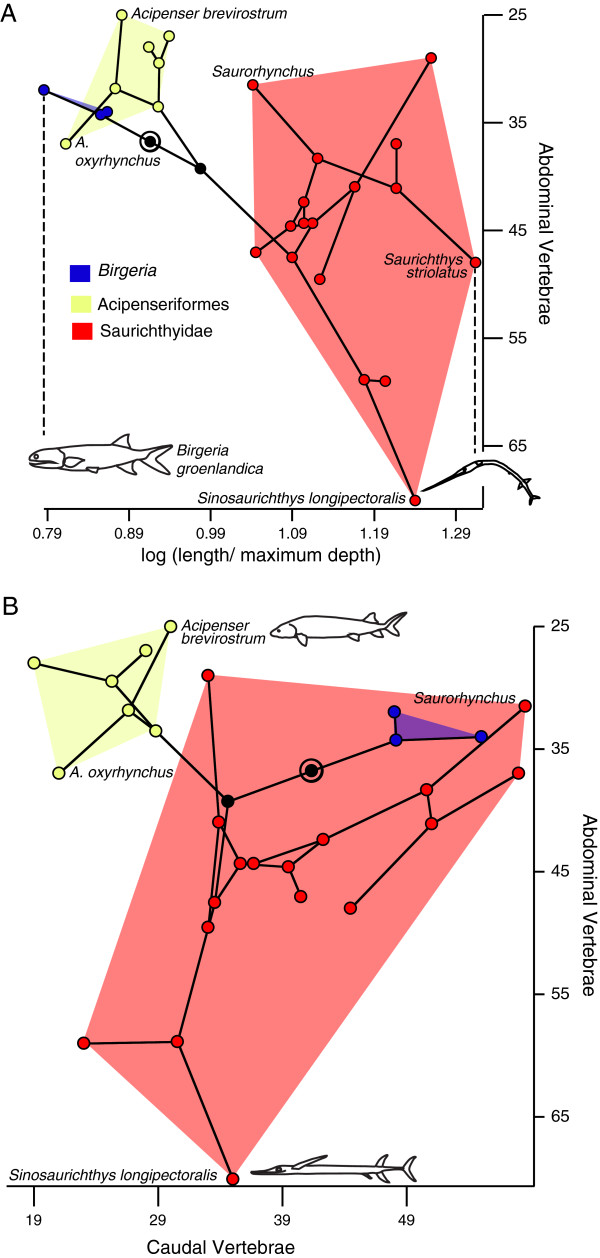
**Phylomorphospace occupation in chondrosteans. A**, Relationship between the number of abdominal vertebrae and fineness ratio. **B**, abdominal and caudal vertebral numbers. Nodes along the backbone of the tree are indicated in black unless they are within one of the indicated higher taxonomic units; the root is indicated by a bullseye. Terminal taxa are represented by nodes connected only to a single branch; hypothetical ancestral states are represented by nodes connected to at least three branches. The placement of the hypothetical ancestral nodes was calculated using weighted squared change parsimony.

There was no significant relationship between the fineness ratio (FR: Figure 2a) and abdominal (r = 0.1, P = 0.37; df = 11; Figure 3a), or caudal (r = 0.26, P = 0.19; df = 11) vertebral counts. There was also no relationship between the number of abdominal and caudal vertebrae (r = 0.17, P = 0.30; df = 11; Figure 3b). As reconstructed with squared change parsimony, only 1.5 vertebrae are added between the outgroups and Saurichthyidae, and there is only a slight increase in body elongation at this node (from an FR value of 12.2 to 13.3) – the stouter body plans seen in *Birgeria* and Acipenseriformes are reconstructed as being secondarily derived from a more elongate ancestral form. *Saurichthys striolatus* was the most elongate form included (Figure 2a), whereas the relatively deep-bodied and brevirostrine forms *Saurorhynchus* and *S. macrocephalus* were among the least elongate forms in Saurichthyidae. All saurichthyids were more elongate than any acipenseriform or *Birgeria* (Figure 3a).

### Lepisosteiformes

**Figure 4:**
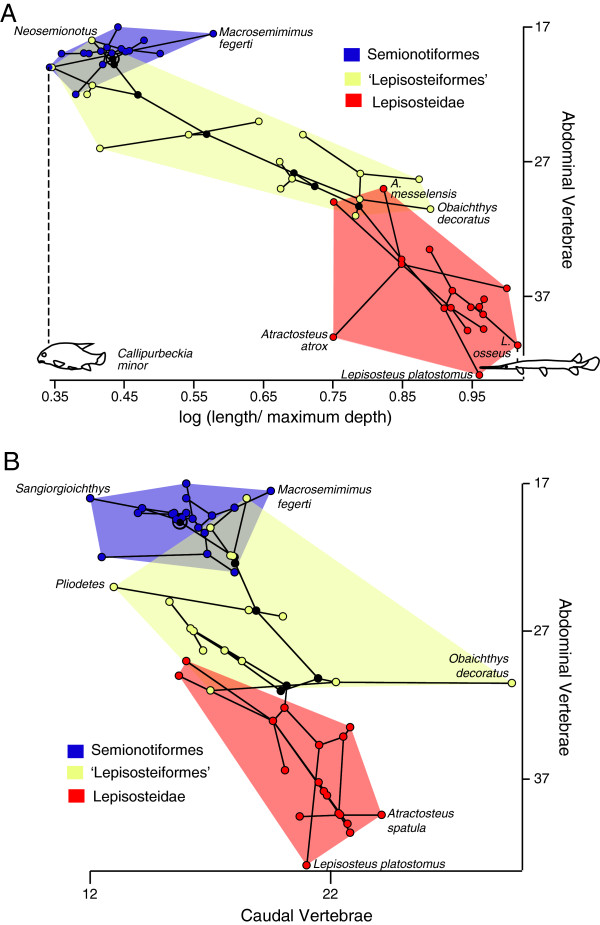
**Phylomorphospace occupation in ginglymodians. A**, Relationship between the number of abdominal vertebrae and fineness ratio. **B**, abdominal and caudal vertebral numbers. Nodes along the backbone of the tree are indicated in black unless they are within one of the indicated higher taxonomic units; the root is indicated by a bullseye. Terminal taxa are represented by nodes connected only to a single branch; hypothetical ancestral states are represented by nodes connected to at least three branches. The placement of the hypothetical ancestral nodes was calculated using weighted squared change parsimony.

Based on independent contrasts, the relationship between the number of abdominal vertebrae and FR is significant (r = 0.56, P = 0.01; df = 14; Figure 4a), but the relationship between the number of caudal vertebrae and fineness ratio is not (r = 0.4, P = 0.07; df = 14). There is no significant correlation between the number of abdominal and caudal vertebrae (r = 0.32, P = 0.09; df = 14; Figure 4b). When specific nodes are examined, of the 7.2 vertebrae added in Lepisosteiformes, 64% are abdominal. A similar trend is observed at the node Lepisosteidae, where in spite of a decrease in the total number of vertebrae by 0.6, the number of abdominal vertebrae increases by 0.8. The number of caudal vertebrae shows relatively low levels of variation in Lepisosteiformes. Only one species, *Obaichthys decoratus*, shows an elevated number of caudal vertebrae, at 30. The total vertebral count for *O. decoratus*, from which the caudal value was calculated, was an estimate based on a single specimen [28]. *Atractosteus spatula* has the second longest caudal region of the sampled species at 24.8 (range: 22–29 [28]). Given the range of caudal vertebral counts observed in *A. spatula* and relative undersampling of the species *O. decoratus*, it is prudent to avoid too much interpretation into potential caudal elongation in *Obaichthys*.

### Esociformes

**Figure 5:**
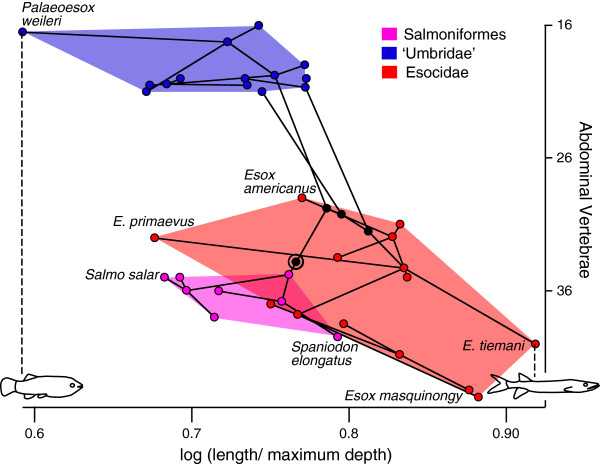
**Phylomorphospace occupation in Esociformes.** Relationship between the number of abdominal vertebrae and fineness ratio. Nodes along the backbone of the tree are indicated in black unless they are within one of the indicated higher taxonomic units; the root is indicated by a bullseye. Terminal taxa are represented by nodes connected only to a single branch; hypothetical ancestral states are represented by nodes connected to at least three branches. The placement of the hypothetical ancestral nodes was calculated using weighted squared change parsimony.

Based on independent contrasts, the relationship between abdominal vertebrae and FR is significant (r = 0.52, P = 0.01; df = 13; Figure 5), that between caudal vertebrae and axial elongation is not significant (r = 0.03, P = 0.43; df = 13), and that between the number of abdominal and caudal vertebrae is also not significant (r = 0.33, P = 0.05; df = 14). Although not significant, the PIC regression of the number of abdominal vertebrae on caudal vertebrae has a slope of 2.91, indicating that the relative increase in the number of abdominal vertebrae is greater than the increase in number of caudal vertebrae (Table 1; Figure 2b).

**Table 1:** Summary of results

	**log FR-abdominal**			**log FR-caudal**			**Abdominal-caudal**		
	**r**	**Slope**	**P**	**r**	**Slope**	**P**	**r**	**Slope**	**P**
Chondrosteans	0.1	0.007 (0.001-0.03)	0.37	0.26	0.009 (−0.04-0.03)	0.19	0.17	0.86 (0.08-3.69)	0.3
Lepisosteiformes	0.56	0.03 (0.01-0.04)	0.01	0.4	0.03 (0.005-0.04)	0.07	0.32	0.98 (0.29-3.19)	0.09
Esociformes	0.52	0.01 (0.01-0.04)	0.01	0.03	0.04 (0.03-0.13)	0.43	0.33	2.91 (1.88-9.99)	0.05
Beloniformes	0.61	0.02 (0.01-0.02)	<0.01	0.4	0.03 (0.02-0.04)	<0.01	0.57	1.84 (1.21-2.23)	<0.01

Values in parentheses represent the 95% confidence interval of the slope. FR = fineness ratio.

### Syngnathiformes

**Figure 6:**
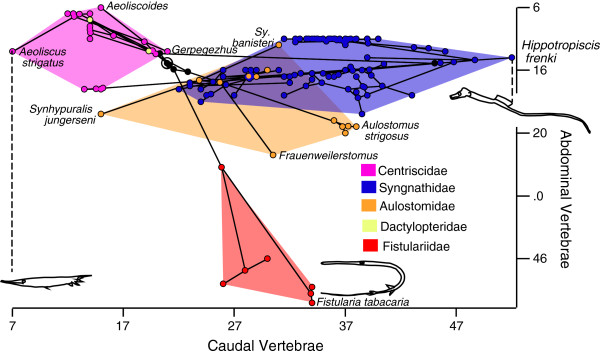
**Phylomorphospace occupation in Syngnathiformes in relation to abdominal and caudal vertebral numbers.** Nodes along the backbone of the tree are indicated in black unless they are within one of the indicated higher taxonomic units; the root is indicated by a bullseye. Terminal taxa are represented by nodes connected only to a single branch; hypothetical ancestral states are represented by nodes connected to at least three branches. The placement of the hypothetical ancestral nodes was calculated using weighted squared change parsimony.

Frequent large increases in total vertebral number are observed (*Syngnathus*, *Hippocampus abdominalis* and *H. bleekeri*, *Hippotropiscus*, *Aulostomus, Fistularia*), and in general large increases in the number of caudal vertebrae are more common than increases in the number of abdominal vertebrae, with the exception of *Fistularia* in which the reverse is true (Figure 2b). The elongate piscivorous ambush predator *Fistularia* is typified by the addition of many more abdominal than caudal vertebrae (19.9 vertebrae added at base of Fistulariidae, of which 15.6 are abdominal [78%]). Based on the available data, Aulostomidae adds only 4.6 vertebrae of which 1.7 are abdominal (37%); therefore most of the added vertebrae are caudal. Based on independent contrasts, the positive relationship between the number of abdominal and caudal vertebrae is significant (r = 0.24, P = 0.01; df = 64), however the strength of the correlation is weak (Figure 6).

### Beloniformes

**Figure 7:**
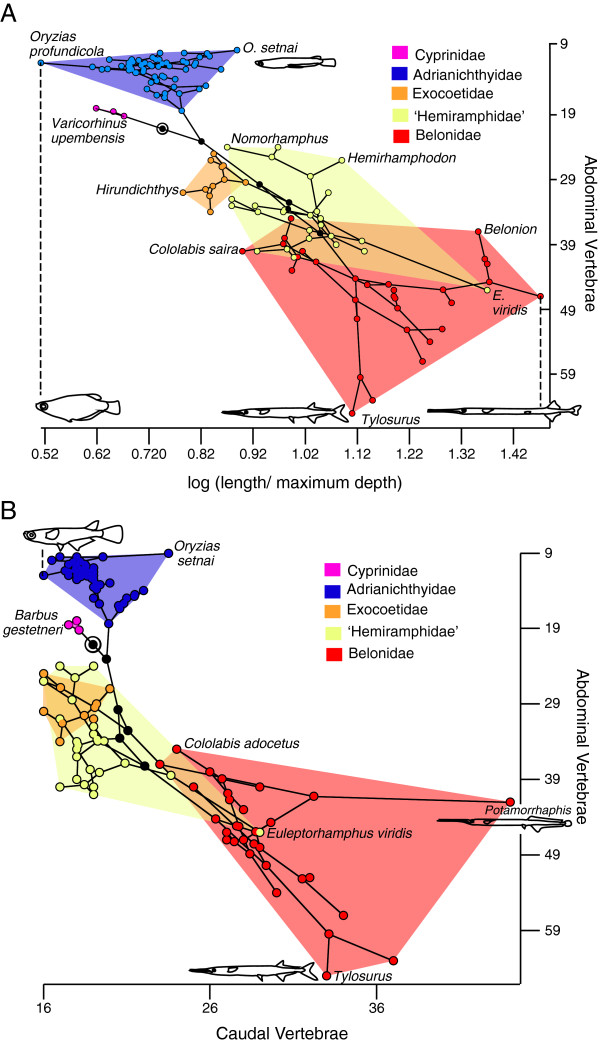
**Phylomorphospace occupation in Beloniformes. A**, Relationship between the number of abdominal vertebrae and fineness ratio. **B**, abdominal and caudal vertebral numbers. Nodes along the backbone of the tree are indicated in black unless they are within one of the indicated higher taxonomic units; the root is indicated by a bullseye. Terminal taxa are represented by nodes connected only to a single branch; hypothetical ancestral states are represented by nodes connected to at least three branches. The placement of the hypothetical ancestral nodes was calculated using weighted squared change parsimony.

Based on independent contrasts, the relationship between the number of abdominal vertebrae and log FR is highly significant (r = 0.61, P = 1.42 × 10^-8^; df = 66; Figure 7a); that between the number of caudal vertebrae and FR is also significant but the correlation is not as strong (r = 0.4, P = 3.30 × 10^-4^; df = 66), and that between the number of abdominal and caudal vertebrae is also significant and positive (r = 0.57, P = 2.33 × 10^-7^; df = 66; Figure 7b). The slope of log FR vs. abdominal vertebral count is 0.02, whereas the slope of log FR vs. caudal vertebral count is 0.03, suggesting that in Beloniformes, a greater number of the additional vertebrae in elongate forms are abdominals than are caudals (Table 1; Figure 2b) (as noted by [3] through the direct examination of abdominal and caudal vertebral counts). However, when raw data are analyzed with partial correlations to untangle this relationship, only the association between the number of abdominal vertebrae and fineness ratio remains significant (r^2^ = 0.82, P = 2.553 x 10^-8^).

### Sphyraenidae

**Figure 8:**
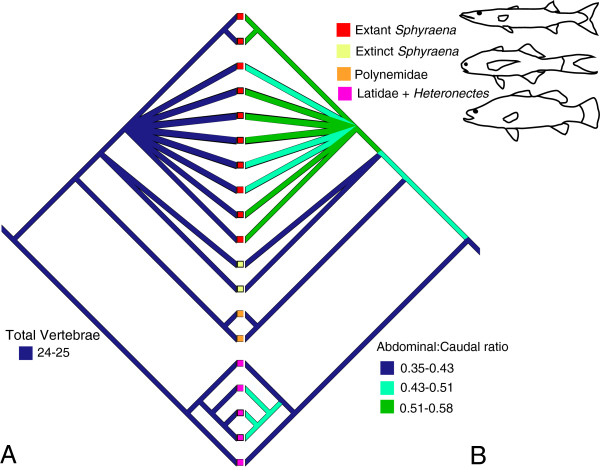
**Variation in total vertebrae relative to the ratio of abdominal vertebrae to caudal vertebrae in Sphyraenidae. A**, Total vertebral count. **B**, ratio of abdominal vertebrae to caudal vertebrae. Ancestral states were reconstructed using squared change parsimony.

Within-clade vertebral counts are very conservative (24–25) in all except derived Pleuronectiformes (Figure 8a). In addition, the number of abdominal vertebrae is invariant, being fixed between 10 or 11 in most taxa, even in derived pleuronectiforms. A notable exception is in extant species of *Sphyraena,* which increase the abdominal vertebral count to 12 or more at the expense of the caudal vertebral count (Figure 8b).

## Discussion

### Preferential addition of abdominal vertebrae

This is the first study to combine analysis of body shape and vertebral number in a comparative context across a broad range of living and fossil species that are hypothesized to share a similar ecomorphotype. Our data suggest that preferential addition of abdominal vertebrae occurs in 5/7 of ‘ambush predator’ clades surveyed (Lepisosteidae, Esocidae, Fistulariidae, Belonidae, Sphyraenidae). Although this characterizes a simple majority of clades examined in this study, it is not statistically significant. In addition, we explicitly tested the relationship between vertebral numbers and body shape in four clades (chondrosteans, Lepisosteiformes, Esociformes, Beloniformes). These clades are only distantly related, and span actinopterygian phylogeny from Neopterygii to Percomorpha. Surveyed species and genera include both living and fossil exemplars. In three of the four clades, the addition of abdominal vertebrae was significantly correlated with fineness ratio, an approximation of axial elongation. This is particularly interesting because one of the more notable features of the ‘ambush predator’ body shape is the lengthening of the skull via an elongated rostrum, which might be thought to increase the fineness ratio without involving any change to the axial skeleton. However, a more elongate skull and a more elongate body do not appear to be independent [7]. For instance, lepisosteids with proportionately reduced skull lengths such as *Masillosteus* and *Cuneatus cuneatus*[29] also show reduced vertebral counts. Likewise, in Beloniformes the less elongate flying fishes are nested within the halfbeaks, and the less elongate sauries are nested within the needlefishes. FR values for these groups are very similar to patterns of rostral elongation [30,31], but a correlation between elongation and regional vertebral numbers is also well-documented [3]. Previous studies have reported that cranial and postcranial elongation appear to be largely independent [4,32], suggesting that their co-occurrence in Beloniformes and Lepisosteiformes may be related primarily to similar selection pressures (e.g., [21]) rather than shared developmental underpinnings.

The degree of axial elongation across Lepisosteiformes and Esociformes, as measured by FR, is variable, and is significantly correlated with the number of abdominal vertebrae but not with the number of caudal vertebrae. However, the two clades differ in that all extant lepisosteids are more elongate (Figure 2a) and most also have higher average abdominal vertebral counts than their fossil relatives, whereas some fossil esocids are more elongate than extant forms (Figures 2a, 5) [33]. Abdominal count is also significantly correlated with FR in Beloniformes; however unlike in esocids and lepisosteids caudal vertebral counts are also significantly correlated with FR. An increase in abdominal vertebral count characterizes the more inclusive clade Beloniformes, but an increase in both abdominal and caudal vertebrae is restricted to Belonidae, suggesting a possible overall increase in somitogenesis in this family. Two outliers are particularly notable for large increases in the number of caudal vertebrae: the adrianichthyid *Oryzias setnai*, and the freshwater belonid *Potamorrhaphis* (Figure 7b). In *Potamorrhaphis*, it is plausible that the high caudal count is linked to the anteroposterior expansion of the median fins [34]; a similar mechanism has been suggested in Gymnotiformes [7]. Abdominal vertebrae are also preferentially added in Fistulariidae, and although no increase in total vertebral number is observed in Sphyraenidae, the number of abdominal vertebrae increases at the expense of caudal vertebrae (homeotic transformation: [6]).

The two clades showing no preferential increase in abdominal vertebral numbers, Saurichthyidae and Aulostomidae, are remarkable exceptions. Both are characterized by limited to no increase in total vertebral numbers relative to outgroups, and thus any correlation between regional vertebral numbers and an elongate body form would, by necessity, arise through a homeotic transformation in which the abdominal–caudal transition is displaced posteriorly, as in Sphyraenidae. However, in neither Saurichthyidae nor Aulostomidae is this observed. Rather, dissociation between the anus/osteological boundary between the abdominal and caudal regions and the anterior insertion of the anal fin [27,35,36] is observed such that a posterior shift in the dorsal and anal fins may occur without a change in the number of abdominal vertebrae. This creates a novel anterior caudal region, in which vertebrae have a caudal identity [37] but lie anterior to the anal fin and are morphologically distinct from more posterior caudal vertebrae (e.g., [38]).

There is extensive non-meristic lability in the location of the boundary between the abdominal and caudal regions in both aulostomids and saurichthyids. The Eocene aulostomid *Synhypuralis* is a notable exception within aulostomids, in that it has one species (*S. jungerseni*) which does not have an expanded region of caudal vertebrae anterior to the insertion of the anal fin. However, variation exists within the genus *Synhypuralis*: *S. banisteri* shows an extremely expanded caudal region anterior to the anal fin, as in extant *Aulostomus*[39]. Although the total number of vertebrae in this genus is relatively constant, the range in relative abdominal and caudal vertebral counts between species of *Synhypuralis* is equivalent to that of the entire syngnathiform data set. The Oligocene aulostomid *Frauenweilerstomus* has a similar total vertebral count to extant *Aulostomus*, but has a larger fraction of abdominal vertebrae than the latter; in addition the novel caudal vertebral region is relatively small: there are 29–30 abdominal vertebrae, and the anal fin is located less than six vertebrae posterior to this transition [40]. This implies that there has been an anterior shift of the abdominal–caudal boundary in *Aulostomus* associated with the elaboration of the novel vertebral region, and that within Aulostomidae, the anterior caudal subregion has been derived at the expense of the abdominal region. A consequence of this shift is the creation of an area anterior to the median fins where musculature can be concentrated, either to transmit force to the caudal fin via elongate tendons through a slender caudal peduncle, or to provide space for muscle mass driving oscillation of the median fins, important in slow-speed swimming. In both cases, increased abdominal rigidity is also expected.

In saurichthyids, there are three ways in which the observed variation in regional vertebral number has been achieved. The first involves a posterior homeotic shift of the abdominal–caudal transition towards the anal fin, increasing the number of abdominal vertebrae at the expense of caudal vertebrae, and specifically at the expense of vertebrae in the anterior caudal region [38]. The second mechanism involves an anterior homeotic shift, which increases in the number of caudal vertebrae at the expense of abdominal vertebrae, either based on an anterior shift in the pelvic fin and anal loop (*Saurichthys krambergeri*), or due to the osteological abdominal–caudal transition having become dissociated from the position of the anus, with the body cavity extending ventral to anterior caudal region (as in *Saurorhynchus*[41]). The latter state could arise through posterior migration of the anus during early development [27].

### Dissociation between elongation and axial regionalization

The correlation between vertebral regionalization and body shape is complex, in spite of the commonalities discussed above. The unexpectedly variable relationship between elongation and vertebral regionalization in several fossil species suggests caution be used when interpreting meristic changes and body shape evolution. It has previously been argued that increasing the vertebral aspect ratio may have been more influential than the addition of abdominal vertebrae at the expense of caudal vertebrae in initially generating the elongate body plan in Sphyraenidae [7], and this is supported by fossil data. The Eocene species *Sphyraena bolcensis* and *S. gracilis* are similar in body shape to the extant species of *Sphyraena,* but show a plesiomorphic 10 + 14 vertebral formula [42]. This implies that the homeotic transformation resulting in an increase in abdominal vertebrae is a relatively recent innovation for *Sphyraena*. More detailed study of both fossil and recent species is needed to assess whether the ratio of abdominal to caudal vertebrae is correlated with variable displacement of the anal fin in Sphyraenidae. Similarly, although Eocene and Oligocene aulostomids are characterized by lower vertebral counts (≥38) than extant species (60–63) [39,40], both extinct and extant taxa are elongate. Synarcual development or skull length, both highly variable within the family, have been hypothesized to be driving elongation in fossil forms with lower vertebral counts [39]. Such variability may typify early radiations of clades.

A complex suite of factors influences the evolution of vertebral number in fishes, including phylogenetic relationships, body shape, swimming mode, size, latitude, temperature, salinity, and life history (reviewed by [43]). However, although vertebral numbers have been shown to change in a modular way across the abdominal and caudal regions of the vertebral column [3], the way in which the factors affecting total vertebral number impact regionalization has barely been investigated. Grande [33] speculated that the evolution of higher vertebral numbers in esocids might be related to speciation during periods of climatic cooling (Jordan’s rule), and the relationship between body size, temperature, and speciation has been supported for esocids based on growth and longevity [44], as has the positive relationship between body size and number of vertebrae [45]. However, overall higher vertebral counts correlated with larger body size underlain by speciation during periods of climatic cooling do not explain the significant association between vertebral count and body shape or the preferential addition of vertebrae to the abdominal region. Based on measurable performance differences, both in terms of increased hunting success [20] and suboptimal acceleration performance [46], as well as multiple instances of convergence in distantly related fishes, it seems unlikely that an increasingly elongate body was a neutral by-product of increasing vertebral count based solely on size and temperature. However, this is not to argue that small differences in body shape underpinned by differences in vertebral number related to size and temperature could not subsequently become positively selected.

Factors influencing intraspecific variation in vertebral number in the medaka (Beloniformes: Adrianichthyidae: *Oryzias latipes*) have been most thoroughly investigated. Both heritable and plastic variation within and between populations was found to be higher for abdominal vertebrae [47-49]. These results were interpreted as suggesting that the genetic variation underpinning variation in the number of caudal vertebrae was absent in Beloniformes, constraining possible evolutionary trajectories [48]. In contrast, subsequent studies found differences in vertebral count between inbred lines of *O. latipes* were driven primarily by changes in the caudal region [50]. Our data suggest that while larger increases in abdominal than caudal vertebral number are observed in Beloniformes, caudal vertebral number is not strongly constrained in this group (contra [48]), supporting the results of Kimura and colleagues [50].

### Hypothesis of median fin placement and constraint

The observed tendency to add abdominal vertebrae as a mechanism driving axial elongation in fishes with the ‘ambush predator’ body shape is consistent with the existence of a conserved positioning module involving the association of the boundary between the abdominal and caudal vertebral regions, the anus, and the anterior insertion of the anal fin [26]. Such a conserved module appears to exert a slight constraint on observed directions of morphological change. Under the facilitation hypothesis of modularity, changes resulting in the posterior displacement of the median fins are most likely to affect the abdominal region, an independent module, rather than occurring via dissociation of the module consisting of the boundary between the abdominal and caudal vertebral regions and the median fins. In evolutionary terms, the probability is higher that selective pressure for posterior displacement of the anal fin will result in the addition of abdominal segments – either synchronous with a general increase in total somite numbers, or more likely in a modular way independent of the number of caudal somites. However, the ‘abdominal–caudal transition/median fins’ module is something that can, and has (as per below) become dissociated in some groups.

A morphologically distinct subregion of the caudal module has evolved, apparently independently, in multiple lineages of bony fishes including Aulostomidae and Saurichthyidae, as discussed above, as well as *Birgeria*, *Parasynarcualis*[39]*,* Dercetidae (a clade of elongate Cretaceous fishes) [51], and rhipidistian fishes including *Eusthenopteron* and *Osteolepis*[52]. Sallan [37] interpreted this anterior haemal-arch bearing region as ‘sacral’ in the osteichthyans *Tarrasius* and *Eusthenopteron*, suggesting that a tetrapod-like Hox code underlying regionalization of the axial skeleton is plesiomorphic for jawed vertebrates and secondarily lost in some teleosts, including the developmental model *Danio*. The results presented here suggest that the expansion of this region has evolved several times independently within actinopterygians. Whether this is indeed underlain by a delayed onset of Hox12 expression relative to Hox11 expression in the paraxial mesoderm creating a long anterior caudal region and posteriorly displaced anal fin, as would be the case if these anterior caudal vertebrae bore a sacral identity, remains to be tested. This is a particularly significant question as it directly addresses whether the sacral region in jawed vertebrates is derived from the abdominal or from the caudal region – in *Saurichthys*, for instance, the new caudal region forms a component of the postanal tail, and is therefore caudal in identity.

## Conclusion

In this study we combine analyses of body shape and vertebral number in a broad range of living and fossil ray-finned fishes to explore whether the convergent evolution of the ‘ambush predator’ body plan is associated with predictable changes in the axial skeleton. Specifically, we tested whether the abdominal region is preferentially lengthened through the addition of vertebrae. Our results indicate that abdominal, rather than caudal, vertebrae are added in the majority (5/7) of ‘ambush predator’ clades surveyed (Lepisosteidae, Esocidae, Fistulariidae, Belonidae, Sphyraenidae). Saurichthyidae and Aulostomidae represent an exception to the general rule of adding abdominal vertebrae, and both of these clades show the development of a novel caudal region anterior to the insertion of the anal fin, morphologically differentiated from more posterior caudal vertebrae. The preferential addition of abdominal vertebrae fits with the previously hypothesized existence of a patterning module consisting of the boundary between the abdominal and caudal vertebral regions, and the anterior insertion of the anal fin. These exceptions indicate that dissociation within this module is possible, leading to increased regionalization within the caudal part of the vertebral column. Our findings suggest that modularity in the axial skeleton may facilitate relatively predictable meristic changes associated with selection for a given body shape in fishes.

## Methods

We examined the evolution of elongate body plans in six clades of ray-finned fishes (Saurichthyidae, Lepisosteidae, Esocidae, Belonidae, Sphyraenidae, Aulostomidae and Fistulariidae). These clades were selected based on a combination of similarities in body shape, inferred hunting style, and the availability of data on regional vertebral numbers. Although some fast-swimming pelagic predators are also elongate (e.g., billfish), morphological specializations of the axial skeleton for continuous swimming have the potential to act as confounding factors when making hypotheses regarding the evolution of vertebral numbers. For instance, a rigid trunk and flexible caudal peduncle reduces the cost of continuous swimming, whereas a flexible trunk is more effective for acceleration from a stationary position [46]. For these reasons, we restrict our analysis to a single ecomorphotype.

We reconstructed the evolution of abdominal and caudal vertebral numbers using squared-change parsimony implemented in Mesquite [53]. Counts were obtained from the literature, except where otherwise noted, for a total of 303 species (Additional file 1). Average values were used when multiple counts were available, with the exception of *Saurichthys costasquamosus*, for which only the count derived from the holotype specimen (PIMUZ T 1855) was used. The reason for this stems from the higher-than-expected morphological variability observed in this species [54,55] leading some authors to question the alpha taxonomic framework [55]. In some clades (e.g. Syngnathiformes, Lepisosteiformes, Sphyraenidae), proxies for vertebral count were employed (ring count, scale row count, and larval somite counts, respectively); these assumptions are discussed on a case-specific basis (see below). To investigate the uncertainty in internal node reconstructions (e.g., [56]), 95% confidence intervals were constructed for ancestral state estimates using the APE package [57] in the R platform [58]. However, this package cannot cope with polytomies, so confidence intervals were calculated only for relatively well-resolved topologies (e.g., Beloniformes).

In several of the clades (Esociformes, Lepisosteiformes, Beloniformes), there is large within-clade variation in the degree of axial elongation observed. In these cases, we establish a correlation between the fineness ratio (FR), and the number of abdominal and caudal vertebrae. The fineness ratio is defined here as the relationship between body length and depth [59] (note the departure from the definition of [60], in which it reflects maximum diameter), and was calculated either from images designed for identification purposes – i.e., those in which there was some assurance that the fish was imaged in lateral view, or from published measurements. FR was used, as opposed to newer metrics calculating the exact contribution of various anatomical modifications to elongation (e.g., Axial Elongation Index [3] and Vertebral Shape Index [59]) as it relied on fewer parameters, allowing data to be more easily culled from the literature. Recent fishes are normally figured in lateral view, and fossil fishes are most often preserved in lateral view, allowing for measurement of depth but not breadth. Correlations were made with Felsenstein’s contrasts correlation (after [61]) (PDAP module [62], implemented in Mesquite). Before phylogenetic independent contrasts (PIC) were calculated, each data set was checked for compliance with the assumptions of the Brownian motion (BM) model of evolution that underlies the PIC method. The absolute values of the contrasts and their standard deviations [63,64] were plotted and the relationship was checked for non-significance, which indicates the branch lengths of the chosen phylogeny adequately fit the tip data (e.g., [65]). In a case whereby diagnostic checks failed, model tests were used to assess the fit of BM to the data as compared to other evolutionary models (Ornstein-Uhlenbeck [OU], lambda, kappa). The Akaike Information Criterion (AIC) was used to differentiate between models and check whether BM was the best fit model (=lowest AIC value) (Additional file 1). Model testing was conducted using the ‘geiger’ package [66] in the R platform [58]. For data sets failing diagnostic checks, branch lengths were transformed using different methods to improve the performance of the PIC method [67]. When multiple significance tests were conducted per data set, a sequential Bonferroni correction for multiple comparisons (as per [68]) was employed within data sets, but not across all data sets.

### Chondrosteans

Phylogenetic relationships were based on the hypotheses of [8,69]. Branch lengths were set using constrained ages of terminal taxa based on youngest stratigraphic occurrence; internal nodes within Acipenseriformes were constrained using fossil occurrence data from [70]. Vertebral count data were based on personal observation of specimens, as well as taken from the literature [38,71-73]; polyodontid data was based on illustrations in [70]. Two of the terminal taxa, *Saurichthys krambergeri* and *S. striolatus*, lack ossified haemal spines thus making the identification of the abdominal–caudal transition difficult. In these cases, the separation between the abdominal and caudal regions was determined based on the position of the anal loop. This landmark coincides with the osteological transition in all species referred to *Saurichthys*, and all saurichthyids except *Saurorhynchus*. Based on the findings of [8], counts of neural arch-like elements calculated from saurichthyids were divided by two to obtain the number of embryonic segments in each region, thus standardizing the data to that obtained for *Birgeria* and Acipenseriformes. Fineness ratio values were log transformed prior to analysis. Assumptions of Brownian motion were not violated based on non-significant correlations between the calculated independent contrasts and their standard deviations.

### Lepisosteiformes

Phylogenetic relationships were based on the hypotheses of [28,74]. Ginglymodian fishes have an extensive fossil record, and so branch lengths were determined based on imposed nodal constraints derived from the age and phylogenetic position of fossil taxa. Vertebral count data were taken from [28,75-79]. Vertebral count data were extrapolated from scale-row counts in fossil forms, with the anterior insertion of the anal fin corresponding to the abdominal – caudal transition. This approach is supported by osteological data [28], which indicates that vertebral counts and scale row counts are generally similar, as is also indicated by dissections [80]. Fineness ratio values were log transformed prior to analysis. Due to poor phylogenetic resolution within *Atractosteus*, 95% confidence intervals could not be reconstructed for nodal estimates. Branch lengths based on fossil data met the assumptions of a BM model required for independent contrasts analysis.

### Esociformes

Phylogenetic relationships were based on the hypotheses of [81,82] and references therein, assuming the monophyly of genera and families. Vertebral count data were taken from [33,81,83-86]. Branch lengths were first set by constraining the age of the terminal taxa in the analysis, as well as the first occurrence of Esocidae based on the review of [33]; however this violated assumptions of BM for some variables, though alternate evolutionary models were found to fit the data less well than BM (Additional file 1). Thus, the branch length method of Nee was used to assign branch lengths and avoid violations in BM assumptions. Nee’s branch length transformation is similar to Grafen’s [87] method of rho transformation, but uses the logarithm of the number of species descended from each node to set the node depth (see [88]).

### Syngnathiformes

Phylogenetic relationships were based on the hypotheses of [89-93]; monophyly of genera and families was assumed in cases where a species had not been included in a phylogenetic analysis. Vertebral count data were taken from [27,35,40,42,94-108]. Because there is a 1:1 relationship between rings and vertebrae [97], ring counts were used when vertebral counts were not available. The fineness ratio was not considered for Syngnathiformes because this group is characterized by repeated evolution of elongate body plans resulting in the ambush predator body plan of interest here as well as the highly specialized body plan of seahorses and pipefish, characterized by extreme flexibility in the caudal region. Branch lengths were first set by constraining the age of fossil terminal taxa in the analysis, and minimum age constraints for higher taxonomic units were also added where possible based largely on those genera/families reported from the Eocene deposits of Monte Bolca [39]. However, attempts to constrain node ages in this way violated assumptions of BM, though this model remained the best fit to the data in comparison to alternatives (Additional file 1). Branch lengths were transformed using the branch length method of Grafen [87]. However, the nodal reconstructions presented are based on untransformed branch lengths constrained with the fossil data. Numerous polytomies prevented the construction of confidence intervals.

### Beloniformes

Phylogenetic relationships were based on the hypotheses of [31,109], including 69 taxa (three cypriniforms as outgroup, 29 adrianichthyids, and 37 species or genera of halfbeaks, flying fishes and needlefishes). Vertebral count data were taken from [3,109-111]. Abdominal vertebral counts were log-transformed prior to estimation of the 95% confidence interval on the ancestral state reconstruction, in order to satisfy the model employed by the ACE package. FR values were log transformed prior to the correlation analysis to linearize the data. Assumptions of Brownian motion were strongly violated for abdominal vertebrae and log FR, but not for caudal vertebrae. Model tests revealed BM was the best supported (AIC = 8.21 compared to OU = 10.2) for the abdominal vertebrae data and also for log FR data (AIC = −100.12, compared to OU = −98.16). Various branch length transformations were applied in order to improve the fit of the branch lengths to the tip data, and the best solution was Grafen’s [87] rho (rho = 0.2), which resolved BM violations.

### Sphyraenidae

Phylogenetic relationships were based on the hypotheses of [112,113] and [114,115] for pleuronectiform interrelationships. Vertebral counts were taken from [3,42,113,116-118].

## Abbreviations

PIMUZ: Paläontologishes Institut und Museum, Universität Zürich.

## Competing interests

The authors declare that they have no competing interests.

## Authors’ contributions

EM designed the study, participated in the statistical analysis, and drafted the manuscript. LW participated in the statistical analysis and helped draft the manuscript. Both authors read and approved the final manuscript.

## Supplementary Material

Additional file 1 Taxonomic data sets and AIC information criteria for model testing in variables not meeting the assumptions of Brownian Motion. Click here for file
