# Supplementary material for: Regionalization of the axial skeleton in the ‘ambush predator’ guild – are there developmental rules underlying body shape evolution in ray-finned fishes?
Source: BMC Evol Biol. 2013 Dec 5;13:265. doi: 10.1186/1471-2148-13-265 (PMC3867419; doi:10.1186/1471-2148-13-265)
Supplement: Additional file 1 — Taxonomic data sets and AIC information criteria for model testing in variables not meeting the assumptions of Brownian Motion. [file 1471-2148-13-265-S1.docx]

**Supplementary data**

1. Data sets

1. Saurichthyidae

2. Lepisosteiformes

3. Esociformes

4. Beloniformes

5. Syngnathiformes

6. Sphyraenidae

1. AIC information criteria for model testing in variables not meeting the assumptions of Brownian Motion

Abbreviations:

Age – age of taxon, in millions of years before present

Total – total average vertebral count

Abd – average abdominal count

Cau – average caudal count

FR – Fineness Ratio

log FR – log-transformed fineness ratio

1. Saurichthyidae

Species Age Total Abd. Cau. FR log FR

*Birgeria stensioei* 242 89.0 34.0 55.0 7.3 0.863

*Birgeria groenlandica* 252 80.0 32.0 48.0 6.1 0.785

*Acipenser brevirostrum* 0 55.0 25.0 30.0 7.6 0.881

*Acipenser oxyrhynchus* 0 58.0 37.0 21.0 6.5 0.813

*Psephurus* 0 55.0 27.0 28.0 8.7 0.940

*Polyodon* 0 47.0 28.0 19.0 8.2 0.914

*Saurichthys curionii* 240 81.0 44.3 36.7 12.7 1.104

*Saurichthys striolatus* 226 84.5 48.0 36.5 20.6 1.314

*Saurichthys krambergeri* 207 95.0 37.0 58.0 16.5 1.217

*Saurichthys paucitrichus* 242 60.0 29.0 31.0 18.2 1.260

*Saurichthys costasquamosus* 242 82.5 49.5 33.0 13.3 1.124

*Saurichthys macrocephalus* 240 78.5 45.0 33.5 11.1 1.045

*Sinosaurichthys longimedialis* 244 82.0 59.0 23.0 16.0 1.204

*Sinosaurichthys longipectoralis* 244 105.0 70.0 35.0 17.4 1.241

*Saurorhynchus brevirostris* 181 90.0 31.5 58.5 11.0 1.041

Topology (Newick format):

((((((S._costasquamosus,S._paucitrichus),(macrocephalus,(S._curionii,((S._striolatus,S._krambergeri),Saurorhynchus)))),((Si._longimedialis,Si._minuta),Si._longipectoralis)),((A._brevirostrum,A._oxyrhynchus),(Psephurus,Polyodon))),(B._stensioei,B._groenlandica)))

1. Lepisosteiformes

Species Age Total Abd. Cau. FR log FR

*Lepisosteus oculatus* 0 58.7 37.2 21.5 9.275 0.967

*Lepisosteus osseus* 0 63.4 40.6 22.8 10.36 1.015

*Lepisosteus platostomus* 0 63.8 42.8 21.0 9.127 0.960

*Lepisosteus bemisi* 50 56.3 33.5 22.8 7.74 0.889

*Lepisosteus platyrhyincus* 0 59.5 37.8 21.7 8.868 0.948

*Atractosteus spatula* 0 63.5 39.4 24.1 9.255 0.966

*Atractosteus tropicus* 0 56.5 36.4 20.1 10.0 1.0

*Atractosteus simplex* 50 45.7 30.0 15.7 5.64 0.751

*Atractosteus messelensis* 48 45.0 29.0 16.0 6.643 0.822

*Atractosteus atrox* 50 62.7 40.0 22.7 5.630 0.751

*Atractosteus tristoechus* 0 60.2 39.5 20.7 8.774 0.943

*Cuneatus cuneatus* 50-56 40.3 25.0 15.3 5.088 0.707

*Cuneatus wileyi* 50-54 45.0 28.3 16.7 7.483 0.874

*Masillosteus janeae* 50 47.3 29.0 18.3 4.732 0.675

*Masillosteus kelleri* 48 43.3 27.0 16.3 4.714 0.673

*Obaichthys decoratus* 110 60.0 30.5 29.5 7.765 0.890

*Dentilepisosteus laevis* 110 48.0 31.0 17.0 6.061 0.783

*Scheenstia zappi* 152.1-153.8 37.0 20.0 17.0 2.22 0.346

*Scheenstia maximus* 152.1-153.8 40.0 22.0 18.0 2.494 0.397

*Macrosemimimus fegerti* 152.1-153.8 37.0 17.5 19.5 3.53 0.578

*Semionotus bergeri* 228.4-237 35.0 19.0 16.0 2.51 0.340

*Semionotus kapffi* 209.4-228.4 33.0 19.0 14.0 2.29 0.360

*Lepidotes minor* 142-145 36.5 20.0 16.5 2.2 0.342

*Lepidotes piauhyensis* 140-150 34.5 22.0 12.5 2.4 0.380

*Neosemionotus* 100.5-113 36.5 18.0 18.5 2.53 0.403

*Pliodetes* 113-126.3 37.0 24.0 13.0 4.4 0.644

*Tlayuamichin* 100.5-111 34.0 18.0 16.0 3.008 0.478

*Semiolepis* 210-220 35.0 17.0 16.0 2.761 0.441

*Sangiorgioichthys sui* 245-244 34.5 19.0 15.5 3.174 0.502

*Sangiorgioichthys aldae* 241.5-237 30.0 18.0 12.0 2.53 0.403

*Thaiichthys* 139.4 46.0 26.0 20.0 2.6 0.415

Additionally, the genus *Lepisosteus* was constrained at a minimum of 75 MYA, *Atractosteus* between 98-103 MYA, Lepisosteidae at 112 MYA, Lepisosteiformes: 125-145MYA

Topology (Newick format):

((((((((((((L._oculatus,L._platyrhyincus),L._osseus),L._bemisi),L._platostomus),(A._tropicus,A._simplex,A._messelensis,A._atrox,(A.spatula,A._tristoechus))),(C._cuneatus,C._wileyi)),(M._janeae,M._kelleri)),(O._decoratus,D._laevis)),(Thaiichthys,Pliodetes)),(Scheenstia_maximus,S._zappi)),Neosemionotus),((S._aldae,Sangiorgioichtyis_sui),((S._bergeri,S._kapffi),(Semiolepis,((*Lepidotes_piauhyensis*),(M._fegerti,(Tlayuamichin,L._minor)))))))

1. Esociformes

Species Age Total Abd. Cau. FR log FR

*Salmo marmoratus* 0 61.0 38.0 23.0 5.179 0.714

*Salmo trutta* 0 59.0 35.0 24.0 4.923 0.692

*Salmo salar* 0 59.0 35.0 24.0 4.816 0.683

*Spaniodon latans*  85 54.5 36.0 18.5 5.215 0.717

*Spaniodon elongatus* 85 57.0 39.5 17.5 6.205 0.793

*Novumbra hubbsi* 0 38.0 20.0 18.0 5.418 0.734

*Novumbra oregonensis* 31 38.5 20.0 18.5 5.929 0.773

*Umbra limi* 0 36.5 20.5 16.0 5.436 0.735

*Umbra prochazkai* 25 36.0 20.5 15.5 4.712 0.673

*Umbra krameri* 0 34.0 20.0 14.0 4.927 0.693

*Palaeoesox fritzschei* 43 31.5 16.0 15.5 5.53 0.743

*Palaeoesox perpusillus* 13.8 36.5 21.0 15.5 4.69 0.671

*Palaeoesox weileri* 24 32.5 16.5 16.0 3.91 0.592

*Boltyshia brevicauda* 54 32.5 19.0 13.5 5.913 0.772

*Dallia pectoralis* 0 41.0 21.0 20.0 5.553 0.745

*Esox* *lucius* 0 60.5 38.5 22.0 6.261 0.797

*Esox* *reicherti* 0 64.5 43.5 21.0 7.524 0.876

*Esox* *masquinongy* 0 65.0 44.0 21.0 7.628 0.882

*Esox* *niger* 0 52.0 33.5 18.5 6.206 0.793

*Esox* *americanus* 0 46.5 29.0 17.5 5.892 0.770

*Esox* *kronneri* 55 47.0 31.0 16.0 6.8 0.833

*Esox* *tiemani* 64 59.0 40.0 19.0 8.288 0.918

*Esox* *primaevus* 33 51.0 32.0 19.0 4.748 0.677

*Esox* *borealis* 25 57.0 35.0 22.0 6.873 0.837

*Esox* *lepidotus* 7 55.0 37.0 18.0 5.625 0.750

Additionally, the genus *Esox* was constrained at a minimum of 73 MYA.

Topology (Newick format):

(((((((E._lucius,E._reicherti),E._masquinongy,E._lepidotus),(E._niger,E._kronneri,E._americanus),E._tiemani,E._borealis,E._primaevus),(Novumbra_oregonensis,Novumbra_hubbsi)),D._pectoralis),(B._brevicauda,(P._fritzschei,P._perpusillus,P._weileri),(U._krameri,U._prochazkai,U._limi))),((Spaniodon_latans,Spaniodon_elongatus),(Salmo_marmoratus,Salmo_trutta,Salmo_salar)))

1. Beloniformes

Species Total Abd. Cau. FR log FR

*Ablennes* 100.0 63.0 37.0 14.09 1.149

*Tylosurus* 98.0 65.0 33.0 12.87 1.110

*Strongylura* 91.0 57.0 34.0 17.6 1.246

*Strongylura leiura* 84.0 52.0 32.0 19.2 1.283

*Xenentodon* 65.0 40.0 25.0 10.34 1.015

*Belone* 84.0 54.0 30.0 18.18 1.260

*Petalichthys* 74.0 47.0 27.0 15.5 1.190

*Petalichthys capensis* 73.0 46.0 27.0 15.5 1.190

*Cololabis saira* 69.0 40.0 29.0 7.93 0.899

*Cololabis adocetus* 59.0 35.0 24.0 9.83 0.993

*Scomberesox simulans* 64.0 38.0 26.0 9.56 0.980

*Scomberesox saurus* 71.0 43.0 28.0 9.87 0.994

*Platybelone* 77.0 48.0 29.0 19.99 1.301

*Pseudotylosurus* 75.0 47.0 28.0 29.6 1.471

*Potamorrhaphis* 86.0 42.0 44.0 23.4 1.369

*Belonion* 60.0 37.0 23.0 22.51 1.352

*Zenarchopterus* 54.0 36.0 18.0 11.22 1.050

*Hemirhamphodon* 42.0 26.0 16.0 12.31 1.090

*Dermogenys* 41.0 24.0 17.0 9.28 0.968

*Nomorhamphus* 43.0 24.0 19.0 7.41 0.870

*Chriodorus* 52.0 33.0 19.0 8.39 0.924

*Hyporhamphus affinis* 57.0 39.0 18.0 12.0 1.079

*Hyporhamphus capensis* 52.0 34.0 18.0 9.5 0.978

*Hyporhamphus improvisus* 48.0 31.0 17.0 11.59 1.064

*Hemiramphus* 60.0 41.0 19.0 9.96 0.998

*Hemiramphus far* 57.0 40.0 17.0 8.47 0.928

*Melapedalion* 53.0 35.0 18.0 11.2 1.049

*Rhynchorhamphus* 59.0 40.0 19.0 13.59 1.133

*Oxyporhamphus* 52.0 33.0 19.0 7.57 0.879

*Oxyporhamphus micropyerus* 51.0 32.0 19.0 7.56 0.876

*Euleptorhamphus viridis* 75.0 46.0 29.0 23.43 1.370

*Parexocoetus* 41.0 25.0 16.0 6.99 0.844

*Fodiator* 42.0 26.0 16.0 6.9 0.839

*Exocoetus* 47.0 27.0 20.0 7.24 0.860

*Hirundichthys* 50.0 31.0 19.0 6.1 0.785

*Prognichthys* 51.0 34.0 17.0 6.88 0.838

*Cheilopogon* 46.0 30.0 16.0 6.96 0.843

*Adrianichthys oophorus*  36.0 15.0 21.0 5.2 0.716

*Adrianichthys poptae* 36.5 16.0 20.5 5.43 0.735

*Adrianichthys kruyti* 36.0 14.5 21.5 6.75 0.829

*Adrianichthys roseni* 36.0 14.0 22.0 5.29 0.723

*Oryzias sarasinorum* 34.0 15.0 19.0 6.55 0.816

*Oryzias bonneorum*  31.5 12.5 19.0 4.96 0.695

*Oryzias nigrimas* 32.5 13.5 19.0 5.89 0.770

*Oryzias orthognathus* 33.0 13.0 20.0 5.56 0.745

*Oryzias nebulosus* 31.0 12.0 19.0 4.86 0.687

*Oryzias timorensis* 30.5 12.5 18.0 4.94 0.694

*Oryzias matanensis* 30.0 12.0 18.0 4.26 0.629

*Oryzias marmoratus* 30.0 12.0 18.0 4.81 0.682

*Oryzias profundicola* 29.0 11.0 18.0 3.25 0.512

*Oryzias celebensis* 30.5 11.5 19.0 5.74 0.759

*Oryzias pectoralis* 30.0 11.0 19.0 6.2 0.792

*Oryzias minutillus* 26.5 10.0 16.5 6.86 0.836

*Oryzias uwai* 26.5 9.5 17.0 6.13 0.787

*Oryzias setnai* 32.5 9.0 23.5 7.75 0.889

*Oryzias javanicus* 29.0 11.5 17.5 4.79 0.680

*Oryzias carnaticus* 29.0 10.5 18.5 4.86 0.687

*Oryzias hubbsi* 27.5 9.5 18.0 5.51 0.741

*Oryzias haugiangensis* 28.0 10.5 17.5 5.49 0.740

*Oryzias dancena* 28.5 10.5 18.0 4.4 0.643

*Oryzias curvinotus* 29.0 11.5 17.5 4.83 0.684

*Oryzias latipes* 29.5 12.0 17.5 6.17 0.790

*Oryzias sakaizumii* 30.0 11.0 19.0 4.8 0.681

*Oryzias luzonensis* 30.0 11.5 18.5 4.65 0.667

*Oryzias mekongensis* 29.0 11.0 18.0 6.45 0.810

*Oryzias songkhramensis* 28.0 12.0 16.0 7.22 0.859

*Oryzias sinensis* 29.0 10.5 18.5 5.41 0.733

*Barbus gestetneri* 36.0 18.5 17.5 4.48 0.651

*Varicorhinus upembensis* 36.0 18.0 18.0 4.15 0.618

Topology (Newick format):

((((((((Ablennes,Tylosurus),((Strongylura,Strongylura_leiura),Xenentodon)),(((Belone,(Petalichthys,Petalichthys_capensis)),((Scomberesox_saurus,Scomberesox_simulans),(Cololabis_adocetus,Cololabis_saira))),(Platybelone,(Pseudotylosurus,(Potamorrhaphis,Belonion))))),((Hemirhamphodon,(Nomorhamphus,Dermogenys)),Zenarchopterus)),(Chriodorus,(Hyporhamphus_affinis,Hyporhamphus_capensis:1.5,Hyporhamphus_improvisus))),(((((Hemiramphus_far,Hemiramphus),(Melapedalion,Rhynchorhamphus)),(Oxyporhamphus_micropyerus_micropterus,Oxyporhamphus)),Euleptorhamphus_viridis),((Parexocoetus,Fodiator),(Exocoetus,(Hirundichthys,(Prognichthys,Cheilopogon)))))),((A._oophorus,((A._kruyti,A._roseni),A._poptae)),(O._sarasinorum,(O._bonneorum,((O._nigrimas,O._orthognathus),(O._nebulosus,((O._timorensis,(O._matanensis,(O._marmoratus,O._profundicola))),(O._celebensis,((O._pectoralis,(O._minutillus,(O._uwai,O._setnai))),((((O._javanicus,O._carnaticus),(O._hubbsi,O._haugiangensis)),O._dancena),(O._curvinotus,(((O._latipes,O._sakaizumii),O._luzonensis),((O._mekongensis,O._songkhramensis),O._sinensis))))))))))))),(V._upembensis,B._gestetneri))

1. Syngnathiformes

Species Age Total Abd. Cau.

*Hippocampus erectus* 0 50.0 11.0 39.0

*Hippocampus reidi* 0 46.0 11.0 35.0

*Hippocampus kuda* 0 47.0 11.0 36.0

*Hippocampus jugumus* 0 49.0 12.0 37.0

*Hippocampus kampelotrachus* 0 50.0 11.0 39.0

*Hippocampus dahli* 0 50.0 11.0 39.0

*Hippocampus planifrons* 0 48.5 11.0 37.5

*Hippocampus biocellatus* 0 47.0 11.0 36.0

*Hippocampus breviceps* 0 51.0 11.0 40.0

*Hippocampus tuberculatus* 0 47.5 11.0 36.5

*Hippocampus taeniopterus* 0 45.5 11.0 34.5

*Hippocampus tristis* 0 47.0 11.0 36.0

*Hippocampus alatus* 0 47.0 11.0 36.0

*Hippocampus queenslandicus* 0 46.5 11.0 35.5

*Hippocampus semispinosus* 0 46.5 11.0 35.5

*Hippocampus abdominalis* 0 57.0 12.5 44.5

*Hippocampus bleekeri* 0 59.0 13.0 46.0

*Hippocampus zebra* 0 49.0 11.0 38.0

*Hippocampus montebelloensis* 0 48.0 11.0 37.0

*Hippocampus whitei* 0 44.5 11.0 33.5

*Hippocampus procerus* 0 45.5 11.0 34.5

*Hippocampus elongatus* 0 44.5 11.0 33.5

*Hippocampus multispinus* 0 46.0 11.0 35.0

*Hippocampus histrix* 0 44.5 11.0 33.5

*Hippocampus hendriki* 0 45.0 11.0 34.0

*Hippocampus angustus* 0 42.5 11.0 31.5

*Hippocampus grandiceps* 0 43.5 11.0 32.5

*Hippocampus barbiganti* 0 44.0 11.5 32.5

*Hippotropiscis frenki* 12 66.0 14.0 52.0

*Amphelikturus dendriticus*  0 53.0 14.0 39.0

*Solegnathus dunckeri* 0 51.5 16.5 35.0

*Cosmocampus elucens* 0 49.5 16.5 33.0

*Syngnathus floridae* 0 51.5 17.5 34.0

*Syngnathus fuscus* 0 57.5 19.5 38.0

*Syngnathus louisianae* 0 51.5 20.0 31.5

*Syngnathus pelagicus* 0 50.5 16.5 34.0

*Syngnathus springeri*  0 61.5 23.0 38.5

*Syngnathus typhle* 0 53.0 17.0 36.0

*Syngnathus abaster* 0 53.0 15.5 37.5

*Syngnathus acus* 0 63.0 20.0 43.0

*Syngnathus nigrolineatus* 0 54.5 16.0 38.5

*Syngnathus rostellatus* 0 55.5 15.0 40.5

*Syngnathus taenionotus* 0 52.5 17.0 35.5

*Syngnathus temminckii* 0 59.0 20.0 39.0

*Syngnathus watermeyeri* 0 55.0 17.0 38.0

*Microphis brachyuros* 0 41.5 18.5 23.0

*Microphis argulus* 0 53.0 16.0 37.0

*Microphis leiaspis* 0 49.0 17.0 32.0

*Microphis fluviatilis* 0 41.0 19.0 22.0

*Microphis mento* 0 43.0 19.0 24.0

*Microphis spinachioides* 0 42.0 16.0 26.0

*Microphis deocata* 0 47.0 16.0 31.0

*Microphis dunckeri* 0 43.0 17.0 26.0

*Microphis cruentus* 0 48.5 17.0 31.5

*Microphis cuncalus* 0 43.0 17.0 26.0

*Microphis manadensis* 0 45.0 21.0 24.0

*Microphis pleurostictus* 0 41.0 18.0 23.0

*Microphis insularis* 0 50.0 18.0 32.0

*Microphis jagorii*  0 44.5 20.0 24.5

*Microphis retzii* 0 44.5 16.0 28.5

*Microphis ocellatus* 0 45.0 16.0 29.0

*Microphis brevidorsalis* 0 44.0 16.0 28.0

*Microphis caudocarinatus* 0 47.0 20.0 27.0

*Solenostomus paradoxus* 0 32.5 19.0 13.5

*Solenostomus cyanopterus* 0 32.5 19.0 13.5

*Solenostomus armatus* 0 34.0 19.0 15.0

*Ramphosus aculeatus* 49 22.0 8.0 14.0

*Ramphosus rosenkranzi* 49 23.5 9.5 14.0

*Eurypegasus* 0 19.0 7.0 12.0

*Pegasus* 0 21.0 7.0 14.0

*Fistularia corneta* 0 76.0 46.0 30.0

*Fistularia petimba* 0 76.0 50.0 26.0

*Fistularia tabacaria* 0 87.0 53.0 34.0

*Fistularia commersoni* 0 84.5 50.5 34.0

*Dactylopterus volitans* 0 22.0 8.0 14.0

*Dactyloptena* 0 22.0 8.0 14.0

*Aulostomus maculatus* 0 60.0 24.0 36.0

*Aulostomus chinensis* 0 63.0 26.0 37.0

*Aulostomus strigosus* 0 63.0 25.0 38.0

*Eoaulostomus bolcensis* 49 46.0 16.0 30.0

*Eoaulostomus gracilis* 49 46.0 17.0 29.0

*Synhypuralis jungerseni* 49 38.0 23.0 15.0

*Synhypuralis banisteri* 49 43.0 12.0 31.0

*Frauenweilerstomus* 30 60.0 29.5 30.5

*Aeoliscus strigatus* 0 20.0 13.0 7.0

*Aeoliscus heinrichi* 30 19.5 7.0 12.5

*Aeoliscus distinctus* 30 19.5 7.0 12.5

*Aeoliscoides* 49 21.0 6.0 15.0

*Gerpegezhus* 55.8 34.0 13.0 21.0

*Macroramphosus scolopax* 0 23.0 9.0 14.0

*Macroramphosus gracilis* 0 25.0 11.0 14.0

Additionally, the family Fistulariidae was constrained at a minimum of 49 MYA, and Dactylopteridae at 49 MYA.

Topology (Newick format):

(((((((Amphelikturus_dendriticus,(Hippocampus_alatus,(Hippocampus_grandiceps,(Hippocampus_hendriki,Hippocampus_multispinus)),Hippocampus_tristis,(Hippocampus_zebra,Hippocampus_montebelloensis),Hippocampus_jugumus,Hippocampus_semispinosus,(Hippocampus_biocellatus,Hippocampus_dahli,Hippocampus_planifrons),Hippocampus_kampelotrachus,(Hippocampus_barbiganti,(((Hippocampus_abdominalis,Hippocampus_bleekeri),((Hippocampus_tuberculatus,Hippocampus_breviceps),Hippocampus_taeniopterus)),(((Hippocampus_procerus,Hippocampus_whitei),(Hippocampus_histrix,(Hippocampus_elongatus,Hippocampus_angustus))),(Hippocampus_erectus,(Hippocampus_queenslandicus,(Hippocampus_kuda,Hippocampus_reidi))))))),Hippotropiscis_frenki),(Syngnathus_fuscus,(((Syngnathus_floridae,Syngnathus_louisianae),Syngnathus_pelagicus),((Syngnathus_temminckii,Syngnathus_watermeyeri),(Syngnathus_acus,(Syngnathus_nigrolineatus,(Syngnathus_rostellatus,(Syngnathus_abaster,(Syngnathus_taenionotus,Syngnathus_typhle))))))),Syngnathus_springeri)),((Microphis_retzii,Microphis_caudocarinatus,Microphis_brevidorsalis,Microphis_ocellatus),(Microphis_fluviatilis,Microphis_spinachioides,Microphis_mento),((Microphis_deocata,Microphis_dunckeri),Microphis_cuncalus,Microphis_cruentus),(Microphis_argulus,Microphis_leiaspis),((Microphis_manadensis,Microphis_jagorii),Microphis_insularis,Microphis_pleurostictus,Microphis_brachyuros)),Cosmocampus_elucens,Solegnathus_dunckeri),(Solenostomus_paradoxus,(Solenostomus_cyanopterus,Solenostomus_armatus))),((Ramphosus_aculeatus,R._rosenkranzi),(Eurypegasus,Pegasus))),(((((Aulostomus_maculatus,Aulostomus_chinensis),Aulostomus_strigosus),Frauenweilerstomus,(Eoaulostomus_gracilis,Eoaulostomus_bolcensis),(Synhypuralis_jungerseni,Synhypuralis_banisteri)),(Dactylopterus_volitans,Dactyloptena)),((Fistularia_commersoni,Fistularia_tabacaria),(Fistularia_corneta,Fistularia_petimba)))),(((Aeoliscus_strigatus,Aeoliscus_heinrichi,Aeoliscus_distinctus),Aeoliscoides,Gerpegezhus),(Macroramphosus_gracilis,Macroramphosus_scolopax)))

1. Sphyraenidae

Species Total Abd. Cau.

*Alectis* 26.0 10.0 16.0

*Alepes* 24.0 10.0 14.0

*Atropus* 24.0 10.0 14.0

*Atule* 24.0 10.0 14.0

*Campogramma* 24.0 10.0 14.0

*Carangoides* 25.0 10.0 15.0

*Caranx* 25.0 10.0 15.0

*Chloroscombrus* 24.0 10.0 14.0

*Decapterus* 25.0 10.0 15.0

*Elegatis* 24.0 10.0 14.0

*Gnathanodon* 24.0 10.0 14.0

*Hemicaranx* 26.0 10.0 16.0

*Lichia* 24.0 10.0 14.0

*Megalaspis* 24.0 10.0 14.0

*Naucrates* 25.0 10.0 15.0

*Oligoplites* 26.0 10.0 16.0

*Pantolabus* 24.0 10.0 14.0

*Parastromateus* 24.0 10.0 14.0

*Parona* 27.0 10.0 17.0

*Pseudocaranx* 25.0 10.0 15.0

*Scomberoides* 26.0 10.0 16.0

*Selar* 24.0 10.0 14.0

*Selaroides* 24.0 10.0 14.0

*Selene* 24.0 10.0 14.0

*Seriola* 25.0 11.0 14.0

*Seriolina* 24.0 11.0 13.0

*Trachinotus* 24.0 10.0 14.0

*Trachurus* 24.0 10.0 14.0

*Ulua* 24.0 10.0 14.0

*Uraspis* 24.0 10.0 14.0

*Polydactylus* 24.0 10.0 14.0

*Arnoglossus* 48.0 12.0 36.0

*Asterorhombus* 37.0 10.0 27.0

*Bothus* 42.0 10.0 32.0

*Chascanopsetta* 62.0 18.0 44.0

*Chascanopsetta crumenalis* 57.0 17.0 40.0

*Crossorhombus* 37.0 10.0 27.0

*Engyophrys* 41.0 10.0 31.0

*Engyprosopon* 37.0 10.0 27.0

*Grammatobothus* 38.0 10.0 28.0

*Japonolaeops* 55.0 11.0 44.0

*Kamoharaia* 53.0 14.0 39.0

*Laeops* 54.0 12.0 42.0

*Lophonectes* 43.0 10.0 33.0

*Monolene* 48.0 10.0 38.0

*Neolaeops microphthalmus* 51.0 13.0 38.0

*Parabothus* 46.0 10.0 36.0

*Perissias* 40.0 10.0 30.0

*Psettina* 40.0 10.0 30.0

*Taeniopsetta* 42.0 10.0 32.0

*Tosarhombus* 40.0 10.0 30.0

*Trichopsetta* 44.0 11.0 33.0

*Paralichthodes algoensis* 31.0 10.0 21.0

*Heteronectes* 24.0 10.0 14.0

*Eolates gracilis* 24.0 10.0 14.0

*Eolates aquensis* 24.5 11.0 13.5

*Psammoperca* 25.0 11.0 14.0

*Lates* 25.0 11.0 14.0

*Sphyraena* 24.0 12.0 12.0

*Sphyraena argentea* A 24.0 14.0 10.0

*Sphyraena aregentea* B 24.0 13.0 11.0

*Sphyraena barracuda* 24.0 14.0 10.0

*Sphyraena pinguis* 24.0 12.0 12.0

*Sphyraena guachancho* 24.0 14.0 10.0

*Sphyraena ensis* 24.0 12.0 12.0

*Sphyraena japonica* 24.0 12.0 12.0

*Sphyraena sphyraena* 24.0 14.0 10.0

*Sphyraena borealis* 24.0 14.0 10.0

*Sphyraena tome* 24.0 14.0 10.0

*Polynemus multifilis* 25.0 10.0 15.0

*Sphyraena bolcensis* 24.0 10.0 14.0

*Sphyraena gracilis* 24.0 10.0 14.0

Topology (Newick format):

((((Sphyraena,(Sphyraena_aregentea_B,Sphyraena_argentea),Sphyraena_ensis,Sphyraena_borealis,Sphyraena_guachancho,Sphyraena_tome,Sphyraena_japonica,Sphyraena_pinguis,Sphyraena_sphyraena,Sphyraena_barracuda),Sphyraena_bolcensis,Sphyraena_gracilis),(Polynemus_multifilis,Polydactylus)),((Eolates_gracilis,(Eolates_aquensis,(Psammoperca,Lates))),(Heteronectes,(Paralichthodes_algoensis,((((Laeops,(Chascanopsetta,Chascanopsetta_crumenalis)),Monolene),(((Crossorhombus,Lophonectes),Arnoglossus),Bothus)),Grammatobothus,Neolaeops_microphthalmus,Psettina,Kamoharaia,Parabothus,Japonolaeops,Perissias,Tosarhombus,(((Taeniopsetta,(Trichopsetta,Engyophrys)),Asterorhombus),Engyprosopon))))))

AIC criterion values for model testing of elongation data

Beloniformes

abdominal FR log FR (not log)

bm model 8.207332 -100.147 363.3834

ou model 10.2032 -98.158 365.1577

kappa model 10.207 -98.1476 365.3834

lambda model 10.2073 -98.147 365.384

Esociformes

abdominal FR log FR (not log) caudal

ou model 156.1892 -49.77349 70.28112 109.9094

bm model 154.1892 -49.45112 70.4704 107.9094

kappa model 156.1892 -47.77315 72.15427 109.9094

lambda model 156.189 -52.139 67.80219 109.9094

Syngnathiformes

abdominal FR log FR (not log) caudal

ou model 466.1509 -212.1557 -215.7911 461.9962

bm model 464.31 -214.1557 -217.7911 459.9962

kappa model 466.133 -212.6993 -216.112 461.99

lambda model 466.1509 -212.1557 -215.7911 461.996
